# Isolation and Characterization of NpCI, a New Metallocarboxypeptidase Inhibitor from the Marine Snail *Nerita peloronta* with Anti-*Plasmodium falciparum* Activity

**DOI:** 10.3390/md21020094

**Published:** 2023-01-28

**Authors:** Aymara Cabrera-Muñoz, Yusvel Sierra-Gómez, Giovanni Covaleda-Cortés, Mey L. Reytor, Yamile González-González, José M. Bautista, Francesc Xavier Avilés, Maday Alonso-del-Rivero

**Affiliations:** 1 Centro de Estudio de Proteínas, Facultad de Biología, Universidad de la Habana, La Habana 10400, Cuba; 2 Institut de Biotecnologia i de Biomedicina and Departament de Bioquímica, Universitat Autònoma de Barcelona, 08193 Bellaterra, Spain; 3 Departamento de Bioquímica y Biología Molecular, Facultad de Veterinaria, Universidad Complutense de Madrid, Ciudad Universitaria, 28040 Madrid, Spain

**Keywords:** metallocarboxipeptidases, carboxypeptidase inhibitor, slow- and tight-binding inhibitor, marine invertebrate, *Plasmodium falciparum*, antimalarial

## Abstract

Metallocarboxypeptidases are zinc-dependent peptide-hydrolysing enzymes involved in several important physiological and pathological processes. They have been a target of growing interest in the search for natural or synthetic compound binders with biomedical and drug discovery purposes, i.e., with potential as antimicrobials or antiparasitics. Given that marine resources are an extraordinary source of bioactive molecules, we screened marine invertebrates for new inhibitory compounds with such capabilities. In this work, we report the isolation and molecular and functional characterization of NpCI, a novel strong metallocarboxypeptidase inhibitor from the marine snail *Nerita peloronta*. NpCI was purified until homogeneity using a combination of affinity chromatography and RP-HPLC. It appeared as a 5921.557 Da protein with 53 residues and six disulphide-linked cysteines, displaying a high sequence similarity with NvCI, a carboxypeptidase inhibitor isolated from *Nerita versicolor*, a mollusc of the same genus. The purified inhibitor was determined to be a slow- and tight-binding inhibitor of bovine CPA (Ki = 1.1·× 10^−8^ mol/L) and porcine CPB (Ki = 8.15·× 10^−8^ mol/L) and was not able to inhibit proteases from other mechanistic classes. Importantly, this inhibitor showed antiplasmodial activity against *Plasmodium falciparum* in an in vitro culture (IC_50_ = 5.5 μmol/L), reducing parasitaemia mainly by inhibiting the later stages of the parasite’s intraerythrocytic cycle whilst having no cytotoxic effects on human fibroblasts. Interestingly, initial attempts with other related proteinaceous carboxypeptidase inhibitors also displayed similar antiplasmodial effects. Coincidentally, in recent years, a metallocarboxypeptidase named PfNna1, which is expressed in the schizont phase during the late intraerythrocytic stage of the parasite’s life cycle, has been described. Given that NpCI showed a specific parasiticidal effect on *P. falciparum,* eliciting pyknotic/dead parasites, our results suggest that this and related inhibitors could be promising starting agents or lead compounds for antimalarial drug discovery strategies.

## 1. Introduction

Metallocarboxypeptidases (MCPs) are zinc-dependent peptide-hydrolysing enzymes involved in important physiological and pathological processes [1,2,3]. Due to the important functions of these enzymes, their proteolytic activity is controlled in many instances. One of the most efficient control mechanisms is protease inhibitors of natural origins, either organic or proteinaceous [3]. 

In contrast to the wide variety of reported proteinaceous inhibitors of endopeptidases, only a few natural inhibitors of metallocarboxypeptidases have been described to date [4]. Several inhibitors have been isolated from plants, including tomato metallocarboxypeptidase inhibitor (TMCI) and potato carboxypeptidase inhibitor (PCI) [5,6]; invertebrates, such as tick carboxypeptidase inhibitor (TCI) and *Haemaphysalis longicornis* carboxypeptidase inhibitor (HTCI) [7,8], leech carboxypeptidase inhibitor (LCI) [9], *Ascaris suum* carboxypeptidase inhibitor (ACI) from the intestinal parasite *Ascaris suum* [10], *Nerita versicolor* carboxypeptidase inhibitor (NvCI) from the marine mollusc *Nerita versicolor* [11,12], and *Sabellastarte magnifica* carboxypeptidase inhibitor (SmCI) from the marine annelid *Sabellastarte magnifica* [13]; and mammals, including latexin from humans and rats [14,15]. All of these are classified as tight-binding inhibitors against the cognate enzymes. These inhibitors have demonstrated potential in the treatment of several diseases in which carboxypeptidases are involved. For example, PCI and TCI, inhibitors of the TAFI (*thrombin-activatable fibrinolysis inhibitor)* metallocarboxypeptidase, have been used as fibrinolytic agents to treat massive pulmonary embolism [16,17] and acute thrombotic events [18]. In addition, human latexin has exhibited potential as a tumour suppressor [19,20].

On the other hand, the potential of strong inhibitors of metallocarboxypeptidases (PCI, LCI, benzyl succinate) to inhibit the growth of *Plasmodium falciparum* in culture has also been proposed [21]. This property has been related to the occurrence of a unique gene coding for an Nna1-like carboxypeptidase in *P. falciparum* [22,23] belonging to the cytosolic carboxypeptidase subfamily (CCPs and M14C). In addition, it has been shown that these enzymes participate in α-tubulin processing, an important mechanism in the dynamics of microtubules during cell division in parasites [23,24,25]. Therefore, its inhibition could produce a negative effect on parasite development [21]. These findings point to this enzyme as a potentially attractive target to treat parasitic infections. It is worth noting that several unsuccessful attempts have been made to isolate or recombinantly produce the enzyme in an active state. 

Due to the importance of this enzyme and the absence of inhibitors targeting it or its subfamily of metallocarboxypeptidases, the availability of new and specific inhibitors could contribute to the design of novel antimalarials as well as to advances in enzyme–inhibitor structure–function knowledge. In this regard, a clear inhibitory activity was recently detected against the related model enzymes of bovine carboxypeptidase A (bCPA) and porcine carboxypeptidases B (pCPB) in an aqueous extract from the marine mollusc *Nerita peloronta* [26]. In addition, extracts from other marine invertebrates, in particular from three ascidians, were reported to inhibit the growth of *P. falciparum* in vitro [27]. These results suggest that *N. peloronta* extract could be a potential and attractive source of carboxypeptidase inhibitors with antimalarial activity. Taking these elements into account, in this work, we set out to identify, purify, and molecularly and functionally characterize a carboxypeptidase inhibitor from the marine mollusc *N. peloronta* (NpCI) as well as test its ability to arrest the growth of *P. falciparum*, which was shown to be successful.

## 2. Results

### 2.1. Purification of NpCI

**Figure 1:**
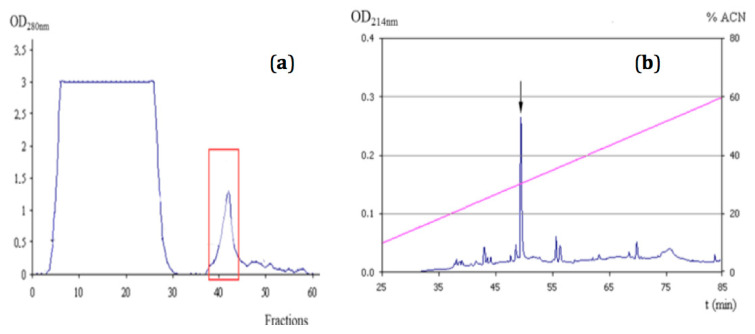
Purification of NpCI from *Nerita peloronta* clarified extract. (**a**) CPA affinity chromatography: column—7.0 × 1.0 cm; linear flow rate—17.0 cm/h. (**b**) RP-HPLC in C8 column with a linear gradient between H_2_O-TFA 0.1% and acetonitrile (ACN)-TFA 0.1% (*v*/*v*) in the 10–60% range: linear flow rate—377 cm/h. The blue line represents absorbance at 280 nm (**a**) or 214 nm (**b**).

The inhibitor NpCI was purified until homogeneity from the mollusc extract treated with 2.5% TCA. The TCA-treated extract showed a total inhibitory activity of 45.15 U and specific activity of 0.3 U/mg towards bCPA. A chromatogram depicting the purification using a CPA–ethyloxy–*Sepharose* CL–6B matrix is displayed in Figure 1a. The elution of bound proteins, achieved by increasing the pH to 12.0, was observed in a single protein small peak, in which the inhibitory activity was concentrated. This chromatography allowed us to recover 80% of the inhibitor applied and obtain a 13-fold purification grade. The active fractions were pooled and applied to an RP-HPLC chromatography C8 column, giving rise to a main symmetric peak with CPA inhibitory activity (Figure 1b). Overall, a purification grade of 103-fold was achieved, with a yield of 78.6%. 

A summary of the purification procedure is shown in Table 1.

### 2.2. Molecular Characterization of NpCI

**Figure 2:**
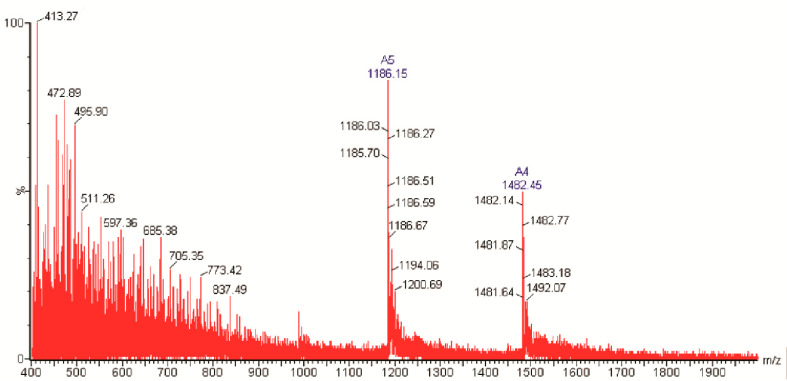
ESI-MS intact mass spectrum analysis from the RP-HPLC NpCI fraction.

A mass spectrometry analysis allowed for the complete molecular characterization of purified NpCI in terms of its molecular mass, number of free and linked cysteines, and full-length amino acid sequence. The ESI-MS spectra obtained for native NpCI showed signals for the multicharged ions of the inhibitor (Figure 2). Signals A5 and A4 corresponded to ions with five and four positive charges, respectively, which allowed for the derivation of the mass of native NpCI as 5925.84 Da, calculated as the average from the signals of the different ions. This value is in agreement with the major signal obtained in the analysis of the active fractions from affinity chromatography. It should be noted that this value was refined afterwards with a precisely tuned and calibrated mass spectrometer to exact mass values of 5921.557 Da and 5927.604 Da for the native and reduced forms, respectively. 

**Figure 3:**
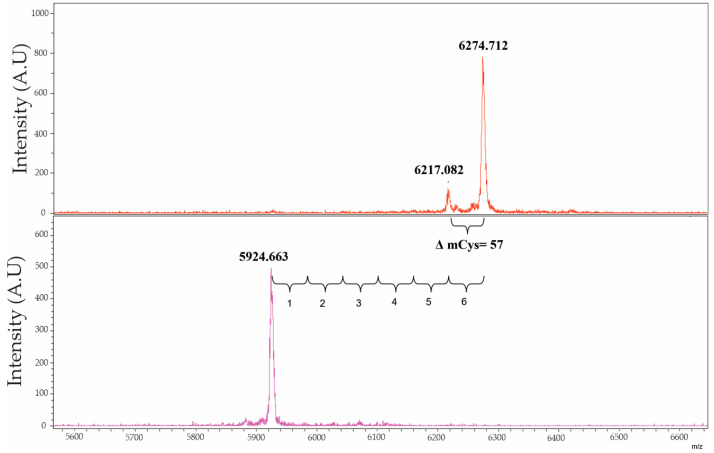
MALDI-TOF mass spectrum of NpCI in the carbamidomethylated and native forms (top and bottom panels, respectively). Samples were treated with 2,5-dihydroxyacetonephosphate (DHAP) as matrix.

In addition, the mass spectrometry analysis of a reduced and carbamidomethylated sample showed an increase of 350.1 Da in the molecular mass of the inhibitor versus the unmodified inhibitor (Figure 3). This fits with the presence of six disulphide-linked Cys residues in the protein, confirmed in the native and reduced forms through analysis with Ellman’s reagent.

**Figure 4:**
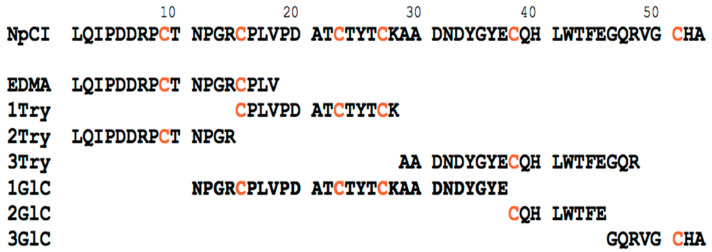
Partial sequences and assembly of NpCI, derived using different experimental approaches. The N-terminus sequence was derived via automated Edman degradation (EDMA) and extended through the LC.ESI-MS/MS sequencing of tryptic peptides (1+2+3 Try) and Glu-C (1+2+3Gl C)-selected endoproteinase-cleaved peptides, as shown in the figure. Cysteine residues are displayed in red. The derived putative 53-residue full protein sequence of NpCI (shown at the top) was validated by fitting its mass with the exact mass of native and intact NpCI derived through a high resolution/high accuracy analysis using MALDI-TOF MS.

Subsequently, the protein sequence of NpCI was derived via the combined analysis of its N-terminal sequencing by automated Edman degradation and the generation of peptides with different proteases, followed by RP-HPLC purification and LC.ESI-MS/MS analysis, which were carried out on both the isolated peptides and the mixtures. The use of trypsin and Glu-C proteinases produced various sets of peptides. The MS/MS analyses of their sequences gave rise to a full coverage of the NpCI sequence, from residues 1 to 53, as shown in Figure 4. This sequence was finally validated through high resolution/high accuracy MALDI.MS analysis using a properly calibrated Bruker timsTOF fleX mass spectrometer, with exact masses of 5921.557 Da (±0.002Da) and 5927.604 Da (±0.002 Da) for the native and reduced forms (mono-charged species), respectively. These data fit exactly with the additive masses of the NpCI residues within its putative (now derived) full sequence, as well as with the state of its six cysteine residues in their disulphide-linked forms.

**Figure 5:**
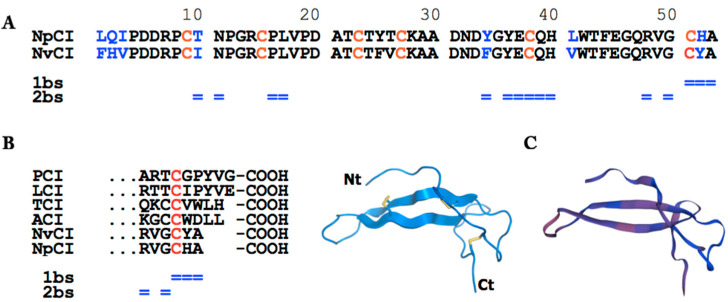
NpCI sequence and conformational analysis. (**A**) NpCI and NvCI sequence alignment. Cysteine residues are highlighted in red, substitutions in blue. The primary and secondary binding sites (1bs and 2bs, respectively) of NvCI to M14 metallocarboxypeptidases [11] are indicated (= sign). (**B**) The boundary between the compact central region and the short C-terminal tail is detected at the last cysteine (in red), as shown by the sequence alignment of the C-tails of NpCI and other proteinaceous carboxypeptidase inhibitors: PCI—the inhibitor from potato [5]; LCI—from leeches [9]; TCI—from ticks [7]; ACI—from *Ascaris suum* [8]; NvCI—from *Nerita versicolor* [11]. (**C**) Cartoon-like representation (left) of the conformation of NvCI from its crystal structure [11] and modelled folding of NpCI (right) displaying a central compact region, a long N-tail, and a short C-tail. Disulphide bonds, represented in yellow for NvCI (left), are most likely the same in NpCI.

As shown in the comparative alignment in Figure 5, the derived full sequence was highly similar to that of NvCI, a carboxypeptidase inhibitor from the marine snail *N. versicolor* of the same genus, which was previously sequenced and characterized by one of our groups [12]. Differences were detected at residues 1–3, 10, 34, 41, and 52—that is, 7 residues of 53 (86.8% of homology)—most of them corresponding to conservative substitutions. The maintenance of the six cysteine residue positions in the two homologous inhibitors, most likely keeping the same disulphide pattern detected in NvCI, is noteworthy. 

A comparison of the C-terminal tail of NpCI with that of other described carboxypeptidase inhibitors is shown in Figure 5b. Low similarities were detected among the inhibitors (with the exception of the NpCI/NvCI pair) regarding both the conservation of residues and tail sizes, which ranged from two to five residues. This merits discussion given that it is an important region including the primary and part of the secondary binding site [11]. 

Furthermore, conformational modelling using *Swiss-Model* [28] and the two available crystal structures (of protein complexes) containing NvCI, used as high-homology templates [11,29], showed that modelled NpCI was characterized by a central compact region (two antiparallel β strands), a long N-tail, and a short C-tail (Figure 5c). The obtained model was compatible with the same disulphide bond pairings as NvCI.

### 2.3. Kinetic Characterization of NpCI

For an in-depth characterization of the inhibition of bCPA and porcine carboxypeptidase B (pCPB) by NpCI, the minimum time required for the establishment of the enzyme–inhibitor equilibrium was determined. The incubation of the enzymes and the inhibitor, at a fixed concentration, for 3, 5, 10, 12, and 30 min before the assay showed that there were statistically significant differences in inhibition between 3 and 10 min. However, after a 10 min incubation, the inhibitory activity did not change significatively, indicating that this was a sufficient time for the establishment of the enzyme–inhibitor equilibrium; it was therefore selected for the following experiments. These results allowed us to qualify NpCI as a slow-binding inhibitor of bCPA and pCPB.

**Figure 6:**
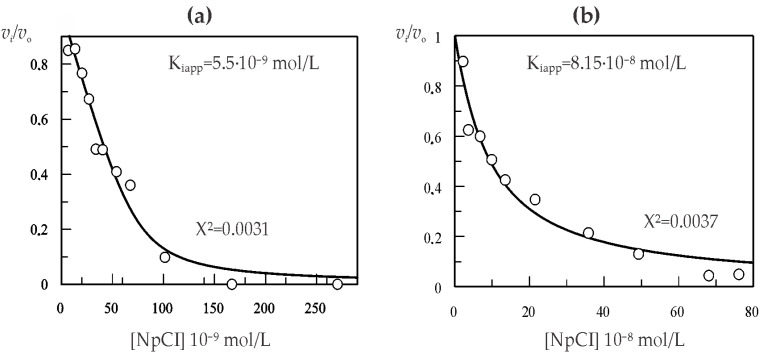
Kinetic characterization of NpCI. (**a**) K_i_ value determination against bCPA. (**b**) K_i_ value determination against pCPB. The fitting of the Morrison equation to the experimental data is displayed, generated using the Grafit program.

Using the 10 min incubation time, the inhibition constants (K_i_) of NpCI against bCPA and pCPB were determined. The experiments were performed under conditions where E_0_/Ki ≤ 10 and [S_0_] = 1K_M_. The fitting of the Morrison equation [30] to the experimental data allowed for the calculation of the *K_iap_* values of 5.5 × 10^−9^ mol/L and 8.15 × 10^−8^ mol/L for the inhibition of bCPA and pCPB, respectively. In both cases, concave curves for the *v_i_/v_0_* vs. [I_0_] plots were obtained, indicating the reversibility of the inhibition (Figure 6a,b). The derived K_i_ values allowed us to confirm that NpCI is a tight-binding inhibitor of bCPA and pCPB under these experimental conditions.

To evaluate the effect of substrate concentration on the inhibitory activity of NpCI, a kinetic analysis was performed with substrate concentrations equivalent to 0.5, 1, and 1.5 K_M_. The increase in substrate concentration in the assay did not produce a statistically significant decrease in inhibitory activity against pCPB. However, a decrease was observed in the bCPA case when the substrate concentration was increased. This result suggests that the substrate can displace NpCI from the active site of the enzyme, which is a characteristic behaviour of competitive inhibitors (data not shown). Based on this behaviour, the K_i_ value of NpCI against bCPA was recalculated considering the substrate concentration and the equation described by Bieth [31] for competitive inhibitors, obtaining a value equal to 1.1 × 10^−8^ mol/L.

After the inhibition of bCPA and pCPB by NpCI, its capability to inhibit serine and cysteine proteases was evaluated. Accordingly, it was determined that NpCI was unable to inhibit porcine pancreatic elastase, bovine pancreatic trypsin, and chymotrypsin, as well as subtilisin A from *Bacillus licheniformis* and papain from *Carica papaya*, with an [I_0_]/[E_0_] rate higher than 200, indicating a narrow specificity of NpCI for metallocarboxypeptidases.

### 2.4. Inhibitory Effect of Purified NpCI on P. falciparum Growth 

Considering that Plasmodium spp. Contain a rather unique cytoplasmic carboxypeptidase [22,23] and having observed inhibitory activity by similar metallocarboxypeptidase inhibitors against this parasite [21], we aimed to evaluate the direct effect of the purified form of NpCI against *P. falciparum* Dd2 (a chloroquine-resistant strain), since in previous assays (not shown) the crude extract of *N. peloronta* was shown to result in the growth delay of *P. falciparum* Dd2 in vitro. In addition, the effect of the inhibitor against the chloroquine-sensitive *P. falciparum* 3D7 strain was also evaluated. 

**Figure 7:**
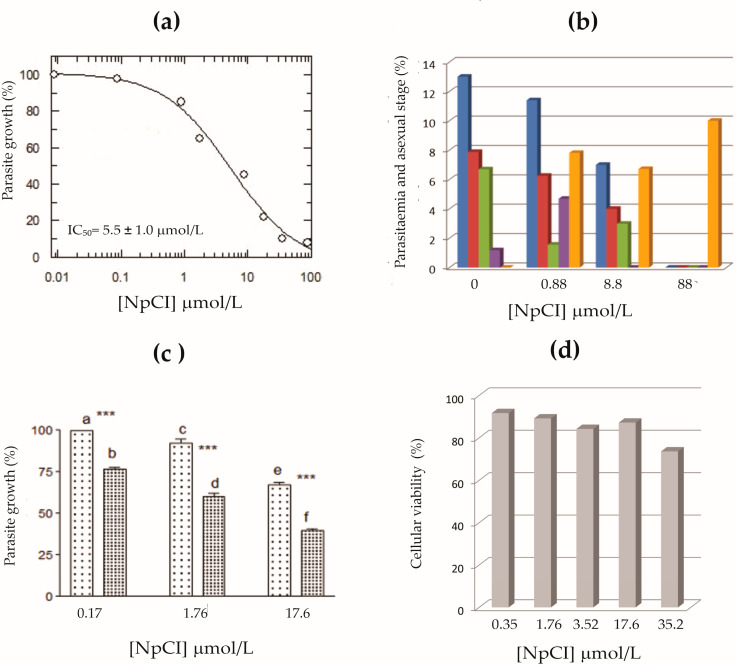
In vitro activity of NpCI in *P. falciparum* and human cells. (**a**) Representative curve of the dose–response effect of NpCI on *P. falciparum* Dd2. Parasites were grown in erythrocyte cultures at 1% initial parasitaemia and incubated with different concentrations of inhibitor. The DNA content was determined at 48 h by microfluorimetry. (**b**) Effect of NpCI on the life cycle of *P. falciparum* Dd2. The presence of different stages of the parasite was observed in culture. Bars indicate percentages of total parasitaemia (dark blue), ring stage (red), trophozoite stage (green), schizont stage (violet), and pyknotic bodies (orange). Data were obtained from the microscopic observation of Wright’s-stained smears from cultures treated for 48 h with different concentrations of NpCI. Percentages were calculated from counts of at least 1000 erythrocytes. (**c**) Comparison of the in vitro inhibitory activity of NpCI for two strains of *P. falciparum*, 3D7 (left) and Dd2 (right), at identical NpCI concentrations. Letters indicate differences for *p* ≤ 0.001, according to post-ANOVA Tukey–Kramer test. (**d**) Cell viability of human fibroblast culture (1BR3G) during 24 h treatment at different NpCI concentrations. For each condition, the percentage of viable cells was determined and plotted.

The purified form of NpCI showed antiplasmodial activity in a dose-dependent manner. Increasing the inhibitor concentration clearly reduced parasite growth (Figure 7a). An IC_50_ value of 5.5 μmol/L was obtained for the NpCI fraction against *P. falciparum* Dd2. The chloroquine control used in parallel resulted in IC_50_ values for both *P. falciparum* strains tested Dd2 and 3D7 within the expected range (0.175 and 0.016 µmol/L, respectively), validating the inhibition assay. 

The effect of the inhibitor on the intraerythrocytic stages of *P. falciparum* Dd2’s life cycle was also assessed using microscopy (Figure 7b). For this purpose, the levels of parasitaemia and the forms corresponding to the different life cycle stages, as well as pyknotic parasites, were analysed by microscopy and quantified at different inhibitor concentrations (Figure S1 and Figure 7b). In the absence of NpCI, *P. falciparum* Dd2 showed the highest levels of parasitaemia (12–14%) with parasites in all stages, mainly rings and trophozoites that had completed the cycle at 48 h from ring-synchronized cultures. However, with NpCI, the cycle was significantly delayed, observed as a decrease in parasitaemia and the abundant presence of schizonts even at concentrations below the IC_50_ value. Furthermore, the abundant presence of pyknotic forms demonstrated the parasiticidal effect of NpCI on the parasite. In fact, the highest concentration tested (88 μmol/L) was completely lethal to the *P. falciparum* Dd2 culture, as only pyknotic forms were observed therein (Figure 7b). These results suggest that the inhibition of growth by NpCI occurs mainly during parasite development towards maturation to merozoite, prior to schizont rupture. 

Finally, it was investigated whether the inhibitory effect of NpCI on *P. falciparum* Dd2 was maintained in other non-chloroquine-resistant strains. A comparison between the 3D7 and Dd2 strains (Figure 7c) showed similar inhibitory strength at the concentrations tested, suggesting that its effect may be transferable to other strains of *P. falciparum* through similar mechanisms of delayed maturation.

The above observations suggest the need to evaluate the cytotoxic effect of NpCI on human cells. Importantly, it was determined that NpCI did not significantly affect human fibroblast cell viability (Figure 7d). Even at the highest concentration tested (six times the IC_50_ value obtained for *P. falciparum* Dd2), the percentage of cell lethality was always lower than 25%; thus, NpCI can be considered as non-toxic to this cell line. 

## 3. Discussion

Metallocarboxypeptidases (MCPs) are zinc-dependent enzymes that hydrolyse the C-terminal residue(s) of peptides and proteins. Their activity is tightly regulated due to the important processes in which they are involved. Several MCPs have been linked to chronic human diseases, such as cancer, fibrinolysis, and inflammation, as well as infectious diseases. Consequently, MCPs and their inhibitors have attracted considerable medical interest as potential drug targets [2,3,32,33,34]. 

Marine species constitute an extraordinary source of bioactive molecules, and a number of these molecules have been tested as antimalarials [35], including protease inhibitors [36]. Many protease inhibitors isolated from marine invertebrates are active against serine proteases and, to a lesser extent, cysteine proteases, although inhibitors from other mechanistic classes have also been isolated. Regarding carboxypeptidase inhibitors, only two of proteinaceous character have been previously described in marine invertebrates, one of which belongs to the phylum Mollusca [11,12,13]. Therefore, the latter is still an unexplored field.

As mentioned earlier, previous studies have reported the capacity of both proteinaceous and organic (synthetic) inhibitors of metallocarboxypeptidases to inhibit the growth of *P. falciparum* in culture, indicating antimalarial potential [21]. This effect could be related to the occurrence and role of a unique gene in *P. falciparum* coding for an M14C metallocarboxypeptidase, a subfamily of enzymes involved in tubulin processing, which is an essential process in cell development [23,24,25]. Increasing evidence for such compounds and their capabilities has therefore gained interest in the development of antimalarials. In our case, we aimed to characterize one of these inhibitors, NpCI, isolated from marine snail *N. peloronta* specimens collected on the Cuban Atlantic coast. For NpCI purification, a combination of TCA and affinity chromatography using CPA–ethyloxy–*Sepharose* as a matrix was employed. A highly purified form was produced using this method. The final refinement of purification, when required (as for protein sequencing), was accomplished using C8 RP-HPLC. 

The mass spectrometry analysis of the purified inhibitor revealed that native NpCI had an average molecular mass of 5925.84 Da, as well as masses of 5921.557 Da and 5927.604 Da for the monoisotopic major forms of the native and reduced forms, respectively. These figures fit exactly with those derived from the deduced protein sequence through the application of the peptide/protein calculator on the Expasy server [28] and are in the mass range of most MCP proteinaceous inhibitors described to date. Only SmCI, from the marine worm *S. magnifica*, and latexin, from mammals, have molecular masses higher than 10 kDa (19.7 kDa and 26 kDa, respectively) [5,6,8,9,10,13,14]. The molecular mass obtained here is in agreement with that reported for NvCI, the only carboxypeptidase inhibitor isolated from molluscs until now [11]. Both inhibitors have 86.7% homology.

The amino acid sequence of NpCI, obtained via the combined analysis of its N-terminal sequence using automated Edman degradation and the generation of peptides with distinct proteases, followed by HPLC purification and/or LC.ESI-MS/MS analysis, showed that the protein consisted of 53 residues with a theoretical mass of 5927.61 Da. This result is in accordance with the molecular mass determined using MS, if it is considered that the six Cys residues form disulphide bonds. A list of the peptides generated and sequenced is shown in Table S1 in the Supplementary Materials. The protein has a high percentage of hydrophobic residues (32%, with three Tyr, one Trp, and one Phe), a higher percentage of acidic than basic residues (13% vs. 7%), a notable presence of Pro (9%) and Gly (7.5%), and a theoretical pI of 4.91, when calculated using the Expasy server [28]. It lacks Met and Ser. 

Furthermore, the presence of six cysteines in the structure of NpCI, likely forming disulphide bonds, agrees with the stability shown by this molecule in the drastic pH conditions used during the purification procedure. The presence of numerous disulphide bonds has been reported in all MCP inhibitors. PCI, TMCPI, and NvCI present three disulphide bonds, while LCI has four, TCI/H1TCI have six, and ACI and SmCI contain seven and nine disulphide bonds, respectively [5,6,8,9,10,13,14]. The maintenance of the six cysteine residue positions in the two homologous inhibitors indicates that both proteins likely have the same disulphide pattern and the same overall folding, in which a central globular disulphide-stabilized fold of forty-three residues is flanked by the N- and C-terminal tails [11,12]. The homology modelling performed using the *Swiss-Model* server confirmed these similarities, with excellent fitting parameters obtained both globally and along the chains, as shown in Figure S2 in the Supplementary Materials section.

The comparison of the NpCI C-terminal tail with that of other MCP inhibitors, displayed in Figure 5b, showed that such inhibitors predominantly (but not absolutely) have an aliphatic residue at the P1 site. This site can be allocated at positions 3–4 of the tail, i.e., in ACI/TCI, following the last cysteine residue (after the compact central protein core) and preceding the P1’ site (the last residue in the longest inhibitors, PCI/LCI), which is known to be cleaved after binding to the carboxypeptidase [37]. No such cleavage occurs for the shortest NpCI or NvCI, or for ACI. Regarding the P2 site, it is possibly allocated at positions 1–2 after the last cysteine; it should be noted that the aromatic residues frequently present in NvCI, LCI, TCI, and other MCP inhibitors are replaced by His in NpCI. Interestingly, the 1–2 positions are among the main points for the interaction and inhibitory action of the inhibitor on putative M14 carboxypeptidase targets [11] and constitute part of the primary binding site (Figure 5b,c). Thus, the Tyr/His substitution at the P2 site of NpCI could account for the strong decrease in its capability to bind and inhibit the enzyme in comparison with other MPC inhibitors, i.e., when compared to NvCI, from 1.0·× 10^−12^ to 5.5 ×·10^−9^ mol/L for the bCPA enzyme [11]. The expected potential loss of the two hydrogen bonds established between the tyrosinate group of Tyr52 and the Arg71 and Arg127 residues of bCPA (essential for the activity of this enzyme) could be partially responsible for the change in K_i_ [7,9,11]. Although the C-tail of NvCI is only two residues long, it is nevertheless involved in the primary binding site for the target enzymes [11,29], whilst the secondary binding site extends along the compact central region, as depicted in Figure 5a,b. Presumably, this is also the case for NpCI. 

The functional characterization of NpCI also showed that this molecule is a slow inhibitor of bCPA and pCPB, which was determined by taking into account the time necessary for the inhibitory enzyme equilibrium to be established. This is a common behaviour for the majority of characterized MCP inhibitors [5,6,8,9,10,11,12,13,14] and is likely determined by the conformation that must be adopted by the inhibitor to introduce the carboxyl tail in the active site of the cognate enzyme [30]. In addition, the K_i_ values obtained allow us to classify NpCI as a tight-binding inhibitor against both of the M14 MCPs assayed. Furthermore, the shape of the dose–response curves obtained suggests the reversibility of the protease–inhibitor interaction analysed in our work. These features are common for all proteinaceous MCP inhibitors described thus far and could constitute an advantage for biomedical and biotechnological applications. 

On the other hand, the decrease in bCPA inhibitory activity produced by the increase in substrate concentration suggests that the substrate used here induced protease–inhibitor complex dissociation. This behaviour is characteristic for competitive inhibitors [38]. However, this behaviour did not seem to apply to pCPB inhibition, suggesting that in this case, the inhibitor and the substrate did not share the same binding site as CPA, a characteristic of non-competitive inhibitors. Nevertheless, for inhibitors that bind to the enzyme in a substrate-like way, as has been described for MCP inhibitors, it can be assumed that the dissociation promoted by the substrate at the assayed concentration is not detectable during the assay time [31]. 

It should be noted that the absence of inhibitory activity against proteases from other mechanistic classes points to a high specificity of NpCI. This result is in accordance with the narrow specificity shown by the other MCP inhibitors, described in [5,6,8,9,10,11,12,14], with the exception of SmCI, which is also able to inhibit serine proteases [13].

The in vitro inhibition of *P. falciparum* growth by NpCI suggests the potential occurrence of a new antiplasmodial target in the parasite and also agrees with the initial results obtained for other MCP inhibitors, such as PCI, LCI, and benzyl succinate [21]. Considering that a single gene coding for a carboxypeptidase-like enzyme (PfNna1, Q8I2A6 in UniProt) has been described in the *P. falciparum* genome [22], it may be hypothesized that the observed inhibition of parasite growth by NpCI was due to the inhibition of such enzyme [21], either direct or indirect.

PlasmoDB, a molecular database for Plasmodium spp., shows that the maximum expression of this enzyme occurs after 42 h in the intraerythrocytic phase of the life cycle of the parasite [39]. This fact places the occurrence of enzyme activity at the later schizont stage, in agreement with the observed cessation of the parasitic life cycle and the increase in pyknotic (nuclei-packed, cell death) forms. 

It should be noted that the IC_50_ value determined for NpCI against the *P. falciparum* in culture was in the low micromolar scale (IC_50_ = 5.5 μmol/L), a figure within the accepted ranges for compounds nowadays investigated as novel lead variants of classical antimalarials [40], particularly when they are not cytotoxic. The great similarity of NpCI to its close homologous NvCI, better known functionally and of reported biological endurance and cell penetration capabilities [12,32], add hopes on the feasibility and interest to investigate its mechanism of action regarding Plasmodium. This could shed light on its target for therapeutic purposes, as well as facilitate its redesign into a smaller active mimicking compound, if convenient.

## 4. Materials and Methods

### 4.1. Materials

*Enzymes*: bovine pancreatic metallocarboxypeptidase A (bCPA) (EC 3.4.17.1), porcine pancreatic metallocarboxypeptidase B (pCPB) (EC 3.4.17.2), bovine pancreatic trypsin (EC 3.4.21.4), bovine pancreatic chymotrypsin A (EC 3.4.21.1), porcine pancreatic elastase (EPP) (EC 3.4.21.36), endoproteinase Lys-C from *Lysobacter enzymogenes* (EC 3.4.21.50) were from provided by Sigma-Aldrich Co. (Saint Louis, MO, USA), papain was obtained from *Carica papaya* (EC 3.4.22.2), *Bacillus licheniformis* subtilisin A (SUBTA) (EC 3.4.21.62) were supplied by Calbiochem Novabiochem Corp (La Jolla, CA, USA) and endoproteinase Glu-C (V8 enzyme from *Staphylococcus aureus*) were purchased from Roche Molecular Biochemicals (Basel, Switzerland). 

*Synthetic substrates:* Substrates N-(4-methoxyphenylazoformyl)-l-phenylalanine (AAFP), N-(4-metoxyphenylazoformyl)-l-arginine (AAFR), Benzoyl-l-arginine-p-nitroanilide-HCl (BAPA), 5-N-Succinyl-alanyl-alanyl-prolyl-phenyl-p-nitroanilide and Leu-p-nitroanilide were purchased from BACHEM (Switzerland), while N-succinyl-alanyl-alanyl-alanyl-p-nitroanilide (N-Suc-(Ala)3-pNA) was obtained from Sigma Chemical Company, (Saint Louis, MO, USA). 

*Columns and matrixes:* HPLC column C8 (3.9 × 150 mm) was purchased from *Waters*, (Milford, MA, USA) and Sepharose CL 6B from Cytiva (Washington, DC, USA).

*Plasmodium falciparum strains:* Dd2 (clone MRA-150) chloroquine-resistant strain and 3D7 (clone MRA-102) chloroquine-sensitive strain were obtained from MR4 (ATCC, Manassas, VA, USA).

### 4.2. Methods

#### 4.2.1. Purification of *Nerita peloronta* Carboxypeptidase Inhibitor (NpCI)

*Nerita peloronta* snails were collected in the tropical sea near Havana (Cuba) and validated by the Cuban Oceanographic Institute. The bodies of the snails were removed from the shell, washed with seawater and homogenized in a home blender. The homogenate was centrifuged for 60 min at 2000× *g*, 4 °C (centrifuge Beckman GS-GKR, Brea, CA, USA) and was filtered through glass wool. The obtained extract was treated with trichloroacetic acid (TCA) (2.5% *v/v*) for 1 h at 4 °C, and then centrifuged at 10 000× *g* for 60 min at 4 °C. The supernatant was adjusted to pH 7.0 with 2 mol/L NaOH and extensively dialyzed against distilled water (v:v 1:200, supernatant/water) using membranes with a molecular weight cut-off of 1000 Da (Amicon, Millipore Corporation, Billerica, MA, USA) and kept at −20 °C. The clarified extract (150.5 mg of total protein) was loaded onto a CPA-ethyloxy-Sepharose CL-6B column (1.0 × 7.0 cm, containing 3.5 mg of CPA/mL gel) previously equilibrated with 5 column volumes (Vc) of Tris-HCl 0.02 mol/L, NaCl 0.5 mol/L, pH 7.5 (equilibration buffer). Non-retained proteins were removed by washing the column with equilibration buffer (5 Vc). The elution was performed with Na_3_PO_4_ 0.05 mol/L, pH 12.0 (5 Vc). The whole process was carried out at room temperature and fractions of 3 mL were collected (2 mL along elution). The linear flow rate was fixed at 17.0 cm/h. The process was monitored by absorbance at 280 nm and bCPA inhibitory activity. The active fraction containing NpCI was then applied to a C8 RP-HPLC column (0.39 × 15.0 cm), equilibrated and washed with 0.1% trifluoroacetic acid (TFA) in water (solution A), and the elution was performed with 0.1% v/v TFA in acetonitrile (solution B) using this gradient: 10% of B during 10 min, followed by a linear gradient from 10 to 40% of B over A along 40 min. The flow rate was 0.75 mL/min (377 cm/h) at room temperature. Protein detection was carried out by absorbance at 214 nm and inhibitory activity against bCPA.

*Analysis of Protein concentration:* Protein concentration was determined by the bicinchoninic acid (BCA) chemical method [41] and using bovine serum albumin (BSA) as standard, according to manufacturer instructions.

#### 4.2.2. Molecular Characterization of NpCI

*Molecular mass determination:* Initial LC.ESI-MS mass spectra were obtained using a mass spectrometer with hybrid octagonal configuration (Micromass, Mundelein, IL, USA) with electronebulization Z-spray (nanoESI) as ionization source. The analyser was operated on positive ion mode and calibrated with a reference mix (Reference material: EMsc-02-0910 registered in PPO 4.09.120.98), in a wide mass range (50–2000 Da). ESI–MS spectrum for the intact protein was acquired in a mass range of 400 to 2000 Da.

*Determination of total and free cysteines:* The number of cysteines present in the NpCI structure was determined by the analysis of MALDI-TOF MS spectra for the native inhibitor and the reduced and carbamidomethylated forms of it. For the free cysteine quantification, the native inhibitor was treated with Ellman’s reagent. 

NpCI was denatured by treatment with 6 mol/L guanidinium-HCl, in 0.1 mol/L Tris-HCl, pH 8.0, for 5 min at 100 °C. After cooling to room temperature, DTT (10 mmol/L) was added. The reaction was performed in 0.1 mol/L Tris-HCl, pH 8.0, for 30 min, at 56 °C, with burble of N_2_. Finally, iodoacetamide, 20 mmol/L, was added and incubated at 37 °C for 30 min. Molecular masses of native and modified NpCI were determined by mass spectrometry, using a MALDI-TOF spectrometer Bruker Ultraflex Extreme. Ionization was achieved with a 337 nm pulsed nitrogen laser, and spectra were acquired in the linear positive ion mode applying a 19 kV acceleration voltage. The 2,5-dihydroxyacetone phosphate (DHAP), prepared at 10 mg/mL in trifluoroacetic acid (TFA) 0.1% / acetonitrile (ACN) 30% was used as matrix for the analysis of protein. The alpha-cyano-4-hydroxy-cinnamic acid (CHCA), sinapinic acid (SA) and 2,5-dihydroxybenzoic acid (DHB) matrices were also used in some instances. MS spectra were analysed using Bruker Daltonics Flex Analysis Software v.3.4 (https://bruker-daltonics-flexanalysis.updatestar.com/en). 

Free cysteines were determined by the incubation of native inhibitor (50 μL) with 10 μL of the Ellman’s reagent, 0.2 mol/L, in Na_2_HPO_4_ 0.1 mol/L buffer, pH 8.0 during 5 min. After that, the absorbance at 405 nm was determined in a plate reader (Multiskan EX, Finland). The concentration of thiol groups was calculated using the calibration curve with aqueous solutions of cysteine, with concentrations between 0.05 and 0.6 mg/mL.

*Protein sequence determination of NpCI:* The freeze-dried NpCI sample, approximately 0.4 mg, after purification by C8 RP-HPLC, was unfolded by treatment with 90 μL of LB buffer (4% SDS or 6 mol/L guanidinium chloride, 1mol/L Tris HCl, pH 7.5) and reduced by adding 10 μL DTT (1 mol/L), with a 30 min incubation at 95 °C. After cooling at 25 °C, the sample was diluted 40 times with Urea buffer (UA; 8 mol/L urea, 0.1 mol/L Tris/HCl pH 8.5), until a volume of 4 mL was reached. Then, it was conditioned and alkylated following the FASP (Filter Aided Sample Preparation) digestion protocol [42], on a 3 KDa Amicon centrifugal filter, at 14000 g, for 10 min, at 10 °C, in each buffer exchange. Briefly, it was washed three times with 500 μL of 8 mol/L urea, 0.1 mol/L Tris-HCl, pH 8.5 buffer, and then treated with 0.05 mol/L iodoacetamide, in 8 mol/L urea, 0.1 mol/L Tris HCl, pH 8.5, in the dark, for 20 min, at 25 °C. Afterwards, the sample was washed three times with 100 μL of UA buffer and three times with 100 μL of 20 mmol/L ammonium bicarbonate buffer, pH 8.5, centrifuged at 14000 g for 15 min, at 10 °C, and freeze-dried in aliquots. Digestion with either trypsin or GluC-endoproteinase was performed at enzyme/protein ratios of 5/100, 1/40 and 1/40 (w/w), at 37 °C, for 18, 1 and 4 h, respectively, in 200 mmol/L triethyl ammonium bicarbonate (TEAB) buffer (pH 8.5), following also the FASP-centrifugal protocol. Samples were either freeze-dried or desalted on C4-ZipTips before MS analyses. An aliquot of the reduced-alkylated protein was subjected to eighteen cycles of automated EDMAN degradation (LF3000 Protein Sequencer, Beckman, Germany). MALDI-TOF.MS analyses were performed in a Bruker Ultraflex Extreme equipment, from Bruker Daltonics, using α-CHCA as a matrix to generate peptide mass fingerprints. The peptide fragments were subsequently subjected to CID fragmentation in the spectrometer, using the LIFT-based approaches [43]. Peptide alignment and protein sequence analyses from the fragmentation spectra were performed using the Bruker Daltonics software and Mascot search engine, with incorporation of the EDMAN degradation data. LC.ESI-MS/MS analysis were performed in an Orbitrap Fusion Lumos Tribid spectrometer (Thermo Fisher Scientific, Waltham, MA, USA), using a μ-precolumn Acclaim C18 PepMap100 and a C18 Acclaim PepMap RSLC (nanoViper, Waltham, MA, USA) analytical column, following the details reported in [44]. Validation of the final derived sequence was achieved by high resolution/high accuracy MALDI.MS analysis on a Bruker timsTOF fleX mass spectrometer.

*Sequence analysis*: Database searches of NpCI fragments from LC-ESI.MS/MS were performed using the Proteome Discoverer package (Thermo-Instruments, v2.1, Waltham, MA, USA), with a 1% false discovery rate, as well as PSI-Blast [45], versus the protein sequences of Uniprot and nrdb-95% databases. Search parameters were set at 20 ppm fragment tolerance; cleaving enzyme trypsin or Glu-endo; missed cleavages 1; and residue modifications as TMTsixplex (Nterm, K), carbamidomethyl (C); carboxymethyl (C); deamidation (N, Q), oxidation (M, W) and dehydration (N). The sequences of NpCI with significant homologies were analysed for multiple sequence alignments with the program MEGA version 3.1. The PEAKS program (from BSI, 2016 vs.) was also used for data analysis, classification and presentation. A list of the sets of the generated, productively sequenced and assigned peptides of NpCI is shown in Table S1. The protein sequence data reported in this paper has been deposited and will appear in the UniProt Knowledgebase under the accession number *C0HM66* (for Carboxypeptidase inhibitor in *Nerita perolonta*).

*NpCI 3D modelling:* Conformational modelling of NpCI was performed using the *Swiss-Model* (*Expasy*) server [28] and validated using the same server. The best template detected by the server was 5mrv.1, with code corresponding to a PDB structure of NvCI in complex to CPO enzyme [29], followed by 4a94.1, in complex to CPA4 enzyme [11], showing both high homology and local and global similarity with the templates. Main derived parameters, in the first case, were 0.85 for GMQE and 0.82+/− 0.11 for QMEANDisco Global, further displayed in Figure S2 of Supplementary Information. 

#### 4.2.3. Kinetic Characterization of NpCI

Inhibition assays were performed by preincubating the inhibitor with the enzymes for 10 min, at room temperature, before adding the substrate. The inhibitory activities of bCPA (3.14 ×·10^−8^ mol/L in the assay) and pCPB (1.5·× 10^−8^ mol/L in the assay) were evaluated using the substrates AAFP and AAFR (0.1 mmol/L, ~1K_M_), respectively [46,47]. Hydrolysis of both substrates was followed at 305 nm at 15 s intervals for 3 min, at 25 °C, in a kinetic spectrophotometer UV-1800 (Shimadzu, Kyoto, Japan). The bCPA activity was evaluated in Tris-HCl 0.02 mol/L, NaCl 0.5 mol/L, pH 7.4, while buffer Tris-HCl 0.05 mol/L, NaCl 0.1 mol/L pH 7.5 was used for pCPB. 

The Ki values of NpCI against bCPA and pCPB were determined by measuring the enzymatic residual activity (a = *v*_i_/*v*_0_) at different inhibitor concentrations and using a fixed enzyme (bCPA: 7.24·× 10^−8^ mol/L and pCPB: 2.6·× 10^−8^ mol/L) and substrate concentrations as described above, where *v_i_* and *v_0_* are initial velocities in the presence and absence of inhibitor, respectively. The determination of Ki values was carried out on equilibrium conditions ([E_0_]/K_i_ ≤ 10), using a previously determined preincubation time of 10 min. Ki values were obtained by fitting the experimental data to the equation for tight-binding inhibitors described by Morrison [30] and implemented in GRAFIT software, version 3.01 (England, UK) [48].

In addition, the effect of the substrate concentration in the inhibitory activity of NpCI against bCPA and pCPB was evaluated. Inhibitory assays were developed following the methodology described above but using different substrate concentration (0.5, 1 and 1.5 K_M_). 

Normality and variance homogeneity were assessed by Kolmogorov–Smirnoff and Bartlett tests, respectively. Then, one-way analysis of variance (ANOVA) followed by Tukey–Kramer’s test was performed for determining statistical differences among substrate concentrations. A *p* ≤ 0.05 was considered significant.

Moreover, the inhibitory activity of NpCI was evaluated against bovine pancreatic trypsin, chymotrypsin A, PPE, papain and SUBTA. The assay was monitoring at 405 nm at 15 s intervals for 3 min, at 25 °C in a kinetic spectrophotometer, using buffers and substrates previously described for these enzymes [49,50,51]. 

#### 4.2.4. Analysis of NpCI Fractions on *P. falciparum* Growth

*Plasmodium falciparum cultures: P. falciparum* Dd2 (clone MRA-150) and 3D7 (clone MRA-102) strains were used for this study. Erythrocytes were obtained from type O^+^ human healthy local donors and collected in Vacuette® tubes with citrate–phosphate/dextrose anticoagulant (Greiner Bio-One, Frickenhausen, Germany). A detailed description of parasite culture, inhibitory studies (as done by NpCI) and synchronization methods have been reported previously [52,53]. A PicoGreen microfluorimetric DNA-based assay was used to monitor parasite growth inhibition as described [52]. Parasite morphology was evaluated from replicate experiments by microscopic analysis of Wright’s-stained thin blood smears. Controls without inhibitors or with chloroquine were performed following the same procedure. Inhibition experiments were performed in triplicate at least twice as a reproducibility proof. Changes in growth were estimated by statistical analysis using the Tukey–Kramer’s test to compare significant differences between individual groups. A *p* ≤ 0.05 was considered significant.

#### 4.2.5. Cytotoxicity Effect of NpCI Fractions

Cytotoxicity effect of NpCI was tested in a cell culture system using human fibroblast cell line 1BR3G (American Type Culture Collection (ATCC)). The cells were grown in Dulbecco’s modified Eagle’s medium (DMEM) supplemented with 10% (*v*/*v*) heat inactivated fetal bovine serum, 2 mmol/L glutamine (Life Technologies Inc., Carlsbad, CA, USA), in a highly humidified atmosphere of 95% air with 5% CO_2_, at 37 °C. Growth inhibitory effect was measured in 96-well microplates (3·× 10^4^ cells per well). After 24 h, different concentrations of inhibitor (0.35, 1.76, 3.52, 17.6 and 35.2 μmol/L) were added and the plates were incubated at 37 °C for 24 h. Aliquots of 20 µL of XTT solution were then added to each well. After 5 h, the colour formed was quantitated in a spectrophotometric plate reader at wavelengths of 490 nm and 610 nm. Cytotoxicity experiments were performed in triplicate at least twice as a reproducibility proof. Cell viability was determined using the following equation:

(1) $$\%\;cellular\;viability=\frac{\left(Abs490nm-Abs610\;nm\right)}{(Abs490\;nm\left(control\right)-Abs610\;nm\left(control\right)}*100.$$

## 5. Conclusions

Metalloproteases, including metallocarboxypeptidases (MCPs), are ubiquitous and important enzymes in living organisms, constituting biomedical targets to control diseases and infections. A novel MCP inhibitor, NpCI, was isolated from the marine snail *N. peloronta*, sequenced, and characterized. NpCI folds in three regions: the N-tail, compact central region (stabilised by three disulphides), and C-tail, with 8, 43, and 2 residues, respectively. It behaves as a slow- and tight-binding inhibitor for M14 MCPs. NpCI was shown to arrest the growth of the malaria parasite *P. falciparum* in vitro, which may indicate the existence of a novel antimalarial target related to the functional properties of this MCP inhibitor. 

## Figures and Tables

**Table 1:** NpCI purification and summary.

Step	[Prots] (mg/mL)	Inhibitory Activity (Ut)	Specific Activity (U/mg)	Yield (%)	Purification (Times)
TCA-clarified extract	3.010	0.903	0.300	100	1
Affinity chromatography	0.183	0.724	3.956	80.2	13
RP-HPLC	0.023	0.710	30.87	78. 6	103

## Data Availability

Not applicable.

## Supplementary Materials

The following supporting information can be downloaded at: https://www.mdpi.com/article/10.3390/md21020094/s1, Figure S1: Representative microscopy images of *P. falciparum* Dd2 cultures, incubated or not with different concentrations of inhibitor: at 0.00, control (A); 0.88 (B); 8.8 (C) and 88 (D) μmol/L of NpCI. Images were obtained from observation of Wright’s-stained smears from cultures treated for 48 h with different concentrations of NpCI. Figure S2: Parameters derived from the modelling of NpCI to NvCI crystal structures; Table S1: Sets of NpCI peptides from Trypsin and GluC hydrolysis, selected for sequence building.

## Abbreviations

AAFP: N-(4-methoxyphenilazoformyl)-L-phenylalanine; AAFR: N-(4-metoxyphenylazoformyl)-L-arginine; ACI:* Ascaris summ* carboxypeptidase inhibitor; BAPA: Benzoyl-L-arginine-p-nitroanilide-HCl; BCA: bicinchoninic acid; bCPA: bovine carboxypeptidase A; BSA: bovine serum albumin; CHCA: alpha-cyano-4-hydroxy-cinnamic acid; CPA4: carboxypeptidase A4; CPO: carboxypeptidase O; DHAP: 2,5-dihydroxyacetone phosphate; DHB: 2,5-dihydroxybenzoic acid; EPP: porcine pancreatic elastase; HTCI*: Haemaphysalis longicornis* carboxypeptidase inhibitor*; IC_50_*: Half-maximal inhibitory concentration; *K_i_*: inhibition constant; *K_M_*: Michaelis-Menten constant; LC.ESI-MS: liquid chromatography electrospray ionization tandem mass spectrometric; LCI: leech carboxypeptidase inhibitor; MALDI-TOF MS: matrix-assisted laser desorption/ionization time-of-flight mass spectrometry; MCP: Metallocarboxypeptidase; NpCI: *Nerita peloronta* carboxypeptidase inhibitor; NvCI: *Nerita versicolor* carboxypeptidase inhibitor; PCI: potato carboxypeptidase inhibitor; pCPB: porcine carboxypeptidase B; SA: sinapinic acid; RP-HPLC: reverse phase high performance *liquid chromatography* SmCI: *Sabellastarte magnifica* carboxypeptidase inhibitor; TCA: trichloroacetic acid; TCI: tick carboxypeptidase inhibitor; TFA: trifluoroacetic acid; TFA: trifluoroacetic acid; TMCI: tomato metallocarboxypeptidase inhibitor; Vc: column volumes; *v_0_*: initial velocity for control assay; *v_i_*: initial velocity in the presence of inhibitor.
