# Supplementary material for: Isolation and Characterization of NpCI, a New Metallocarboxypeptidase Inhibitor from the Marine Snail Nerita peloronta with Anti-Plasmodium falciparum Activity
Source: Mar Drugs. 2023 Jan 28;21(2):94. doi: 10.3390/md21020094 (PMC9966942; doi:10.3390/md21020094)
Supplement: Supplementary file 1 [file marinedrugs-21-00094-s001.zip › r2095675-FigS1.new+legend.pdf]

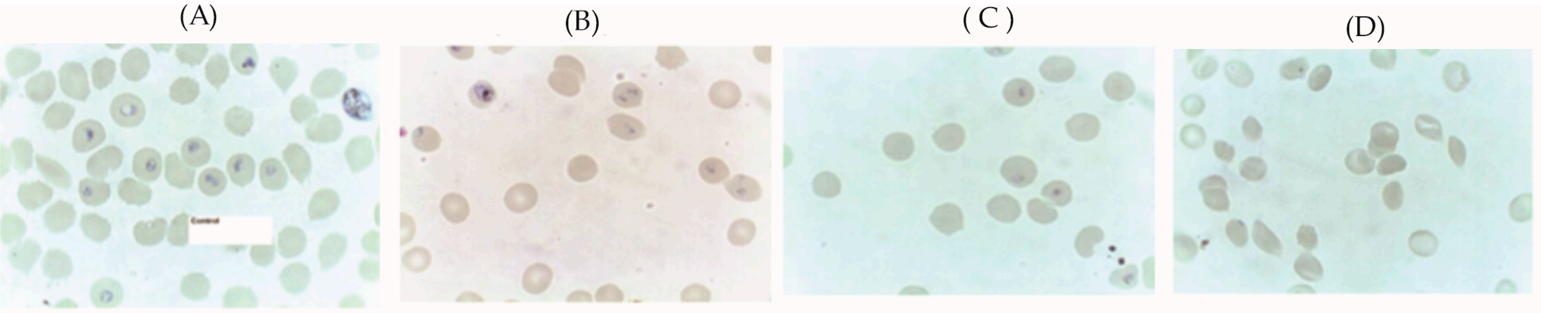

Figure S1: Representative microscopy images of *P. falciparum* Dd2 cultures, incubated or not with different concentrations of inhibitor: at 0.00, control (A); 0.88 (B); 8.80 (C) and 88,00 (D) mol/L of NpCI. Images were obtained from observation of Wright's-stained smears from cultures treated for 48 h with different concentrations of NpCI.
