# Supplementary material for: Isolation and Characterization of NpCI, a New Metallocarboxypeptidase Inhibitor from the Marine Snail Nerita peloronta with Anti-Plasmodium falciparum Activity
Source: Mar Drugs. 2023 Jan 28;21(2):94. doi: 10.3390/md21020094 (PMC9966942; doi:10.3390/md21020094)
Supplement: Supplementary file 1 [file marinedrugs-21-00094-s001.zip › r2095675-FigS2+legend.pdf]

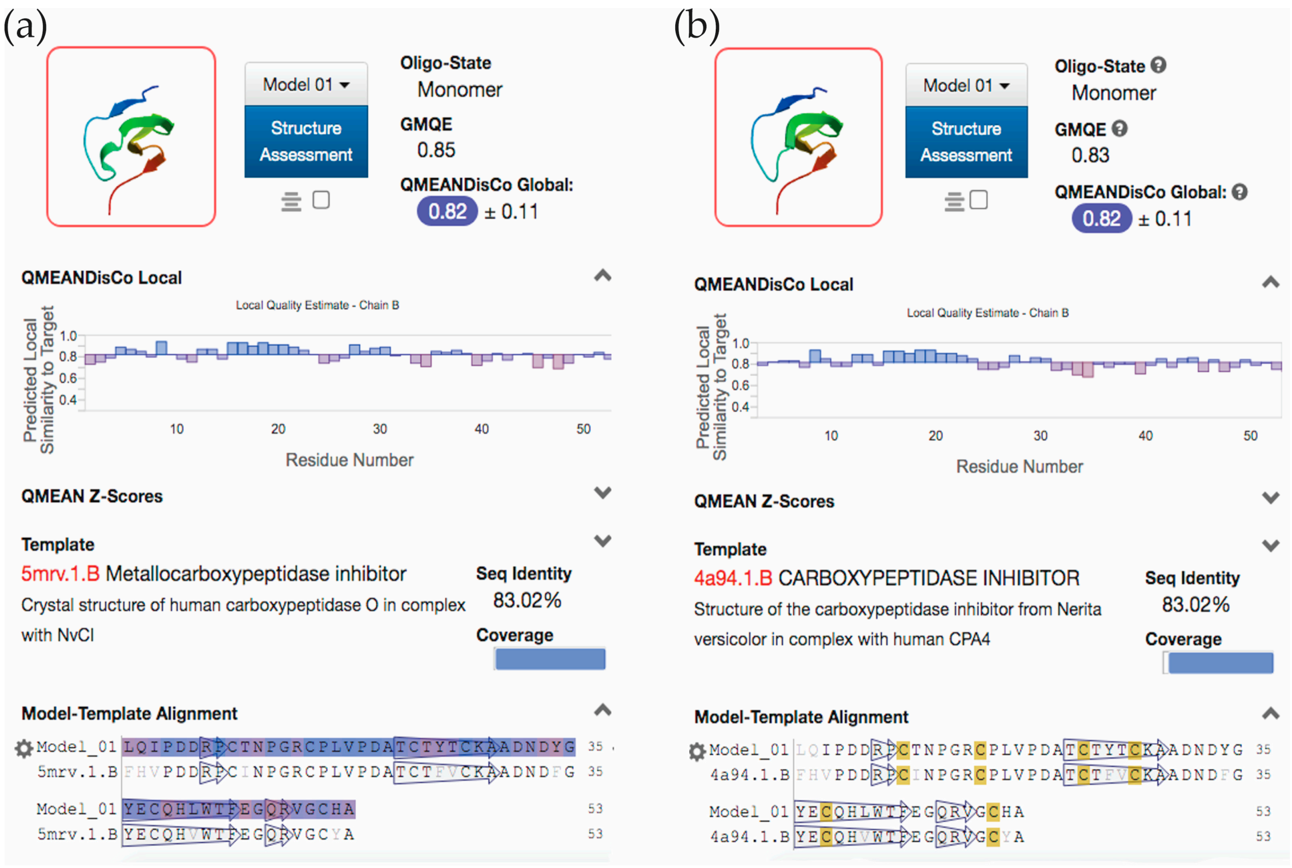

Figure S2. Parameters Derived from the Modeling of NpCI to NvCI Crystal Structures The modeling of NpCI from *Nerita perolonta* was perfomed from its protein sequence in the Expasy/Swiss Modeller server [28], using as templates the structures of NvCI from *Nerita versicolor* taken from the detailed crystal structures of its complexes with (a) Carboxypeptidase O [37], and (b) Carboxypeptidase A4 [11], found at PDB with codes 5mrv.1.B. and 4a94.1.B. The derived results and parameters are shown below.
