# Supplementary material for: Isolation and Characterization of NpCI, a New Metallocarboxypeptidase Inhibitor from the Marine Snail Nerita peloronta with Anti-Plasmodium falciparum Activity
Source: Mar Drugs. 2023 Jan 28;21(2):94. doi: 10.3390/md21020094 (PMC9966942; doi:10.3390/md21020094)
Supplement: Supplementary file 1 [file marinedrugs-21-00094-s001.zip › r2095675-TableS1+legend.pdf]

Table S1. Sets of NpCI Peptides from Trypsin and GluC Hydrolysis, Selected for Sequence Building.

Fraction: mcE71_Tryp_5ng_20220519.raw / Ion Source: ESI(nano-spray) ÷ Fragmentation Mode: CID, CAD (y and b ions) / MS & MS/MS Scan Modes: FT-ICR/Orbitrap. Analytical program, PEAKS DB (2016 vs; BSI-Thermo).
The <Pep nº> column indicates the peptides displayed in the alignments of Fig.4, although all of them were used to derive the sequence.

| **Supporting Peptides** |

Peptides (Trypsin-derived)	-10lgP	Mass	Length	ppm	m/z	z	RT	Scans	Start	End	Pep nº	PTMs
C(+57.02)PLVPDATC(+57.02)TYTC(+57.02)K	115.64	1684.7260	14	1.1	843.3712	2	42.32	1941	15	28	**1**	Carbamidomethyl
C(+58.01)PLVPDATC(+57.02)TYTC(+57.02)K	53.86	1685.7100	14	11.9	843.8723	2	42.40	1946	15	28	**1**	Carboxymethyl (C); Carbamidomethyl (C)
LQIPDDRPC(+57.02)TNPGR	90.67	1637.7943	14	0.0	546.9387	3	38.40	1704	1	14	**2**	Carbamidomethyl(C)
LQ(+.98)IPDDRPC(+57.02)TNPGR	68.86	1638.7783	14	11.1	547.2728	3	38.43	1706	1	14	**2**	Deamidation (N,Q); Carbamidomethyl (C)
AADNDYGYEC(+57.02)QHLWTFEGQR	90.40	2459.0237	20	0.7	820.6824	3	50.79	2449	29	48	**3**	Carbamidomethyl (C)
AADN(+.98)DYGYEC(+57.02)QHLWTF EGQR	53.81	2460.0076	20	7.9	821.0163	3	50.80	2450	29	48	**3**	Deamidation (N,Q); Carbamidomethyl (C)

Fraction: mcE71_GluC_25pg_20220601.raw / Ion Source: ESI(nano-spray) ÷ Fragmentation Mode: CID, CAD (y and b ions) / MS & MS/MS Scan Modes: FT-ICR/Orbitrap. Analytical program, PEAKS DB (2016 vs; BSI-Thermo).
The <Pep nº> column indicates the peptides displayed in the alignments of Fig.4, although all of them were used to derive the sequence.

| **Supporting Peptides** |

Peptides (GluC-derived)	-10lgP	Mass	Length	ppm	m/z	z	RT	Scans	Start	End	Pep nº	PTM
LQIPDDRPC(+57.02)TN	73.38	1327.6190	11	2.8	664.8187	2	31.88	1608	1	11		Carbamidomethyl (C)
TC(+57.02)KAADNDYGYE	61.23	1405.5455	12	1.8	703.7813	2	28.07	1182	26	37		Carbamidomethyl (C)
C(+57.02)QHLWTFE	57.80	1119.4807	8	4.7	560.7502	2	40.96	2466	38	45	**2Glc**	Carbamidomethyl (C)
PGRC(+57.02)PLVPDATC(+57.02)TYTC(+57.02)	56.62	1866.8063	16	-2.0	934.4086	2	35.94	1979	12	27		Carbamidomethyl (C)

GQRVGC(+57.02)HA	52.81	883.4083	8	2.4	442.7125	2	19.94	411	46	53	**3Glc**	Carbamidomethyl (C)
KAADNDYGYE	50.30	1144.4673	10	0.3	573.2411	2	27.45	1114	28	37		
PGRC(+57.02)PLVPD	49.50	1009.5015	9	-0.9	505.7575	2	31.74	1597	12	20		Carbamidomethyl (C)
LQIPDDRPC(+57.02)TNPGRC(+57.02)PLVPD	46.16	2319.1099	20	-5.5	774.0397	3	37.83	2158	1	20		Carbamidomethyl (C)
ATC(+57.02)TYTC(+57.02)KAADN DYGYE	45.37	2001.7721	17	0.3	668.2648	3	30.75	1490	21	37		Carbamidomethyl (C)
C(+57.02)Q(+.98)HLWTFE	42.96	1120.4647	8	0.0	561.2396	2	41.78	2546	38	45	**2Glc**	Carbamidomethyl(C); Deamidation (Q)
KAADN(+.98)DYGYE.C	40.46	1145.4513	10	0.4	573.7332	2	28.12	1188	28	37		Deamidation (Q)
C(+57.02)QHLW(+15.99)TFE	38.03	1135.4757	8	0.3	568.7453	2	38.69	2249	38	45	**2Glc**	Carbamidomethyl(C); Oxidation (W)
LQIPDDRPC(+57.02)TN(-18.01)	37.31	1309.6085	11	-0.8	655.8110	2	32.24	1654	1	11		Carbamidomethyl(C); Dehydration (N)
C(+57.02)QH(+15.99)LWTFE	37.15	1135.4757	8	0.8	568.7456	2	40.78	2450	38	45	**2Glc**	Carbamidomethyl(C); Oxidation (W)
NPGRCPLVPDATCTYTC(+57.02)KAADNDYGYE	31.49	2993.2629	27	-3.1	998.7585	3	37.21	2094	11	37	**1Glc**	Carbamidomethyl (C)
